# Minnesota State Medical Society

**Published:** 1869-05-15

**Authors:** E. J. Davis

**Affiliations:** Rec. Secretary; Mankato, Minn.


### PROCEEDINGS OF SOCIETIES.

### *Minnesota State Medical Society.*

About two years before Minnesota became a State, that is, in December, 1855, a State Medical Society was organized in St. Paul, by adopting a Constitution and By-Laws, and the election of the following officers:

President—Dr. THOS. R. POTTER.

Vice President—Dr. J. H. MURPHY.

Recording Secretary—Dr. J. V. WREN.

Corresponding Secretary—Dr. J. D. GOODRICH.

Treasurer—Dr. DAVID DAY.

Censors—Drs. W. H. MORTON, F. R. SMITH, J. H. STEWART, A. E. JOHNSON and A. E. AMES.

Two subsequent meetings were held, the last being at St. Anthony, in 1857. After that period the Society was allowed to languish. The rebellion broke out, and a large number of its members entered the service, and hence no meetings were held until a call was issued, signed by some of its members and others, for a Convention of Physicians at St. Paul, on the 2d of February, 1869.

Dr. J. H. Murphy, the Vice President of the Society, called the meeting to order, and Dr. D. W. Hand was chosen temporary Secretary.

Dr. A. E. Ames, on behalf of the Censors, proposed the names of forty physicians as duly qualified for membership. They were all accepted.

After some desultory discussion concerning the revival of the old Society or the organization of a new one, it was, by unanimous consent, resolved to re-organize.

The following physicians were recommended, and elected officers of the new Society, for the ensuing year:

President—Dr. SAMUEL WILLY, St. Paul.

Vice President—Dr. A. E. AMES, Minneapolis.

Corresponding Secretary—Dr. A. WHARTON, St. Paul.

Recording Secretary—Dr. E. J. DAVIS, Mankato.

Treasurer—Dr. S. B. SHEARDOWN, Stockton.

Censors—Drs. J. H. MURPHY, St. Paul; N. R. HILL, Minneapolis; W W. MAYO, Rochester; A. C. WEDGE, ALBERT LEA and J. C. RHODES, Stillwater.

The first Tuesday in February was the time chosen for holding the annual meeting, and St. Paul selected as the place.

Dr. O. H. Howitt, of Red Wing, was appointed to read an essay at the next annual meeting.

The Society is, also, to hold semi-annual meetings, in different localities throughout the State, and the first of these meetings is to be held at Owatonna, on the 16th of June. The following programme was accepted as the order of exercises at the next semi-annual meeting.

2 P. M.—Essay by Dr. Kimball, Minneapolis; subject, Rheumatism. Dr. Wharton, St. Paul, and A. B. Stewart, Winona, to introduce the discussion of the subject.

3 P. M.—Subject, Quacking; its Diagnosis and Treatment. Dr. Mattocks, St. Paul, and Dr. C. H. Hewitt, Red Wing, to introduce the discussion of the subject.

7. P. M.—Address by Vice President, and discussion of the subject by the members present.

Drs. J. H. Murphy, St. Paul; C. H. Hewitt, Red Wing, and S. B. Sheardown, Stockton, were appointed delegates to the United States Medical Association.

E. J. Davis, Rec. Secretary.

Mankato, Minn., April 15, 1869.

